# An Individualized Exercise Intervention for People with Multiple Myeloma—Study Protocol of a Randomized Waitlist-Controlled Trial

**DOI:** 10.3390/curroncol29020077

**Published:** 2022-02-07

**Authors:** Jennifer L. Nicol, Carmel Woodrow, Brent J. Cunningham, Peter Mollee, Nicholas Weber, Michelle D. Smith, Andrew J. Nicol, Louisa G. Gordon, Michelle M. Hill, Tina L. Skinner

**Affiliations:** 1 School of Human Movement and Nutrition Sciences, The University of Queensland, Brisbane 4072, Australia; b.cunningham@uq.edu.au (B.J.C.); anic9909@bigpond.net.au (A.J.N.); t.skinner@uq.edu.au (T.L.S.); 2 QIMR Berghofer Medical Research Institute, Brisbane 4006, Australia; louisa.gordon@qimrberghofer.edu.au (L.G.G.); michelle.hill@qimrberghofer.edu.au (M.M.H.); 3 Haematology, Division of Cancer, Princess Alexandra Hospital, Brisbane 4102, Australia; carmel.woodrow@health.qld.gov.au (C.W.); peter.mollee@health.qld.gov.au (P.M.); 4 Faculty of Medicine, The University of Queensland, Brisbane 4006, Australia; 5 Haematology, Cancer Care Services, Royal Brisbane and Women’s Hospital, Brisbane 4006, Australia; nicholas.weber@health.qld.gov.au; 6 School of Health and Rehabilitation Sciences, The University of Queensland, Brisbane 4072, Australia; m.smith5@uq.edu.au; 7 Brisbane Clinic for Lymphoma, Myeloma and Leukaemia, Greenslopes Private Hospital, Brisbane 4120, Australia; 8 Palliative Care Outcomes Centre, School of Nursing and Cancer, Queensland University of Technology, Brisbane 4059, Australia; 9 UQ Centre for Clinical Research, Faculty of Medicine, The University of Queensland, Brisbane 4006, Australia

**Keywords:** exercise, physical activity, multiple myeloma, hematology, adherence, bone lesions, randomized controlled trial, quality of life, cost-effectiveness

## Abstract

People with multiple myeloma (MM) are second only to people with lung cancer for the poorest reported health-related quality of life (HRQoL) of all cancer types. Whether exercise can improve HRQoL in MM, where bone pain and lesions are common, requires investigation. This trial aims to evaluate the efficacy of an exercise intervention compared with control on HRQoL in people with MM. Following baseline testing, people with MM (*n* = 60) will be randomized to an exercise (EX) or waitlist control (WT) group. EX will complete 12-weeks of supervised (24 sessions) and unsupervised (12 sessions) individualized, modular multimodal exercise training. From weeks 12–52, EX continue unsupervised training thrice weekly, with one optional supervised group-based session weekly from weeks 12–24. The WT will be asked to maintain their current activity levels for the first 12-weeks, before completing the same protocol as EX for the following 52 weeks. Primary (patient-reported HRQoL) and secondary (bone health and pain, fatigue, cardiorespiratory fitness, muscle strength, body composition, disease response, and blood biomarkers) outcomes will be assessed at baseline, 12-, 24- and 52-weeks. Adverse events, attendance, and adherence will be recorded and cost-effectiveness analysis performed. The findings will inform whether exercise should be included as part of standard myeloma care to improve the health of this unique population.

## 1. Introduction

Recent remarkable advances in treatments for people with multiple myeloma (MM), an incurable cancer of plasma cells, are contributing to longer life expectancies [1]. However, the inevitable relapse and substantial disease- and treatment-related side effects can reduce patients’ health-related quality of life (HRQoL). In a recent population-wide study, people with MM had the most functional limitations and were second only to lung cancer for the highest psychological distress and lowest quality of life of all cancer types [2]. HRQoL has been shown to correlate with all-cause mortality in all disease stages of MM [3,4,5,6,7].

Exercise has been shown to be an effective therapy for improving HRQoL in other cancer patient populations both during and following treatment [8]. However, whilst these short-term benefits of exercise have been consistently reported, the maintenance of these benefits beyond the completion of interventions is understudied. In people with MM, only one intervention published as a single arm study [9] and later a randomized controlled trial [10] has explored the effects of aerobic and/or resistance exercise on HRQoL. The randomized controlled trial failed to find any effect of exercise on HRQoL. The lack of effect may be explained, at least in part, by low attendance to the prescribed intervention, i.e., once-weekly group classes at a hospital outpatient gym (only 80% attending >50% of sessions) and twice weekly home-based exercise (completion rates not reported). Adherence to the prescribed protocol was not reported for either the supervised or unsupervised sessions. Thus, the exercise dose received was lower than that prescribed and potentially insufficient to elicit benefits to HRQoL. Individualized supervised exercise sessions have been found to result in greater adherence and better outcomes compared with unsupervised/home-based sessions for people with cancer [11]. Therefore, future research is required which investigates the effect of an individualized supervised exercise intervention on the HRQoL of people with MM.

Cancer-related fatigue, decreased physical functioning, and breathlessness are experienced with very high incidence and severity among people with MM, contributing to poor HRQoL [12]. Whilst aerobic exercise has been found to effectively reduce fatigue, enhance physical functioning, and increase cardiorespiratory fitness in other cancer populations [13], several randomized controlled trials [10,14,15] exploring its efficacy on these outcomes in patients with MM found no significant improvements. The aerobic exercise in these studies was prescribed at a moderate intensity. High intensity interval training (HIIT) has been shown to elicit greater improvements in cardiorespiratory fitness in a shorter amount of time compared with moderate intensity aerobic exercise in other populations [16,17]. HIIT has also been found to be as effective as, if not superior to, moderate intensity aerobic exercise for improving body composition [18,19] and fatigue [20], whilst also helping cancer survivors maintain their motivation to exercise [21]. Two studies have investigated the effect of HIIT in people with hematological cancers which included people with MM in the study cohort [22,23]. However, despite showing that it was safe and feasible in this cancer population, there was no separate analysis for MM. Whether HIIT provides a feasible, effective, and time-saving training strategy to enhance cardiorespiratory fitness and physical functioning and reduce fatigue in patients with MM warrants investigation.

Exercising in the presence of bone lesions has been identified as a major safety concern by hematologists [24] and patients [25] alike, particularly during resistance training and impact loading activities. Bone involvement occurs more frequently in MM than in any other cancer [26], with up to 85% of patients with MM developing lytic lesions during the course of their disease [27]. Bone pain is commonly experienced in people with MM, with 45% experiencing bone pain in the vertebrae and 27% experiencing bone pain in other sites, such as the ribs and pelvis [25]. Reductions in bone mineral density and degradation of bone microarchitecture are frequent manifestations in people with MM due to inflammation secondary to myelo-suppressive treatments [28]. Prolonged periods of bed rest after autologous hematopoietic stem cell transplantation, high doses of steroids, nausea, and appetite loss can all have detrimental effects on total body and muscle mass [29,30,31], with the potential to contribute to cancer cachexia. Combined with an older average age at diagnosis (70 years) and high rates of peripheral neuropathy, people with MM are at a greater risk of falls and fractures. Combined resistance training and impact loading have been found to improve bone health, muscular strength, and balance, and thus quality of life in other populations [32,33,34]; further research is required to confirm its safety and efficacy in people with MM.

Whilst the short-term benefits of exercise on HRQoL in cancer survivors have been consistently reported [35], the maintenance of these benefits beyond the completion of interventions is often understudied. A recent systematic review showed that the increases in physical activity during exercise interventions are not maintained six months after intervention cessation in community-dwelling older adults [36]. Conversely, a systematic review of exercise interventions for women with breast cancer [37] found small improvements in physical function and fatigue still evident at long-term (≥6 months) follow-up; albeit only nine studies were found. Further investigation of the long-term maintenance of physical activity and psychosocial health benefits following exercise interventions is clearly required.

We aim to investigate the influence of an individualized supervised exercise intervention on HRQoL in people with MM compared with usual care. The effects of exercise on outcomes that may influence HRQoL (i.e., fatigue, bone pain, cardiorespiratory fitness, bone health, muscle strength, body composition, and balance), as well as the influence of exercise on markers of bone turnover, adipokines, immune functioning, and metabolomics will be compared with usual care. Adverse events, attendance, and adherence will be recorded and a cost-effectiveness analysis performed. Finally, after twelve weeks of supervised exercise and a further twelve weeks of a stepped-down supervision approach, the maintenance of the effect of exercise six months after cessation of all exercise support will be explored. It is hypothesized that a 12-week supervised exercise intervention will improve HRQoL, and factors that influence HRQoL, in people with MM compared with usual care. Exercise is also hypothesized to improve markers of bone turnover, adipokines, immune functioning, and metabolomic profiles compared with usual care. These effects are hypothesized to return to baseline levels six months after cessation of all exercise support.

## 2. Methodology

This trial was prospectively registered on 12 March 2019 with the Australian New Zealand Clinical Trial Registry 12619000387123. (Protocol version 6, dated 19 March 2020) (http://www.anzctr.org.au/Trial/Registration/TrialReview.aspx?id=376687&isReview=true— accessed 1 October 2021). Trial registration includes all components of the World Health Organization Trial Registration Data Set, as recommended by the International Committee of Medical Journal Editors. A SPIRIT (Standard Protocol Items: Recommendations for Interventional Trials) checklist [38] is provided in File S1.

### 2.1. Trial Design and Setting

**Figure 1:**
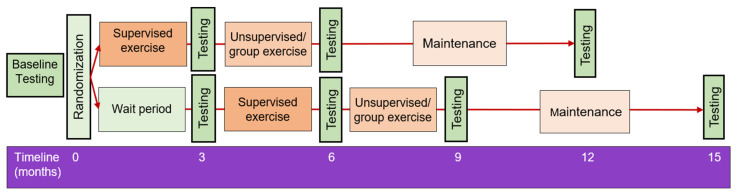
Schematic representation of the study protocol.

The study design is depicted in Figure 1. People diagnosed with MM will be invited to participate in this multi-site, single blind, randomized waitlist-controlled trial. A waitlist control group design was chosen to reduce participant dropout from a non-exercise control group [39]. Participants randomized to the waitlist control group will be encouraged to maintain their customary physical activity during the 3-month wait period.

The exercise program follows a stepped down supervisory approach with reduced supervision after three months. All participants will complete an assessment battery (see Table 1) at baseline, at the end of the supervised phase, and at 6- and 12-months following baseline testing (see Figure 1).

### 2.2. Participants

Men and women diagnosed with multiple myeloma will be invited to participate.

Inclusion criteria

i ≥18 years old;
ii Diagnosis of MM;
iii Free of any musculoskeletal, neurological, respiratory, metabolic, or cardiovascular conditions that may prevent safe completion of the exercise demands of the study;
iv Able to give informed consent; and
v Able to attend participating sites across southeast Queensland, Australia, to complete exercise training sessions and The University of Queensland for the testing sessions.

Exclusion criteria

i Abnormal resting electrocardiogram (ECG) with changes that suggest increased risk of exercise-induced cardiac event;
ii Unstable angina;
iii Cognitive impairment that impedes the ability to complete questionnaires; and
iv Any intellectual or physical disability which would make participation in an exercise intervention unsafe for the individual.

Interested participants will be individually interviewed using a medical history form and will be required to obtain their doctor’s approval prior to participating in the study. Individual participants will be discontinued from the trial, in agreement with their doctor where appropriate, if any major surgery or health condition arises that will significantly affect their safety to participate in exercise for a duration of >one month, or if the participant elects to withdraw their consent for any reason.

### 2.3. Recruitment

Study posters and flyers will be displayed at multiple hematology clinics across Brisbane, Australia. Study co-investigators will directly approach eligible patients with MM. The study will also be advertised via Myeloma Australia and the Leukaemia Foundation support group network for participant self-referral.

Eligible participants will receive the Participant Information Sheet, Participant Consent Form, and Medical Doctor Clearance Form. Their doctor’s written clearance and the signed Participant Consent Form will be collected and securely stored by the principal investigator.

### 2.4. Randomization

Following completion of the baseline testing session, participants will be stratified by disease stage (active treatment, transplant eligible; active treatment, transplant non-eligible; non-active; relapsed) before being randomly assigned (1:1 allocation ratio) to waitlist control (WT) or exercise intervention (EX) group. A research officer independent of the study will perform the randomization using a computerized random number generator. To ensure allocation concealment, a block size of four will be used. Randomization history will be kept secure by the independent research officer in an electronic password-protected file. The allocation result will be communicated in confidence by the independent research officer to the trial’s accredited exercise physiologist (AEP) within 48 h after completing the baseline testing session. The principal investigator will conduct all testing sessions and analyses and be blinded to group allocation. Un-blinding will not be permissible under any circumstances. Due to the nature of the study, neither the AEPs prescribing the training nor participants can be blinded to the randomization assignment.

### 2.5. Exercise Intervention

**Figure 2:**
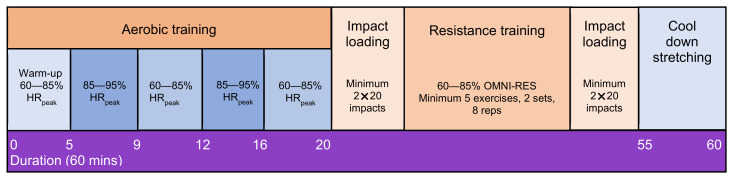
Schematic representation of the 12-week supervised exercise intervention protocol. HR_peak_: peak heart rate; OMNI-RES: OMNI-Resistance Exercise Scale of Perceived Exertion; reps: repetitions; mins: minutes.

Participants will complete the modular multi-modal resistance, aerobic, and impact loading exercise program under the supervision of the trial AEP on two days per week, in addition to one home-based session. Supervised exercise sessions will be offered at multiple locations across southeast Queensland, Australia: North Lakes Health Precinct, Greenslopes Private Hospital, Princess Alexandra Hospital, Royal Brisbane and Women’s Hospitals Hospital, and The University of Queensland. A familiarization session with the trial AEP will be completed in the week prior to commencement to explain the exercise program and guide participants through several exercises that are more technically challenging. The 60 min training session will follow the procedure as described in Figure 2. The exercise intervention is designed to provide an optimal stimulus to the cardiorespiratory, skeletal, and neuromuscular systems while maximizing compliance and retention. The program adheres to the American College of Sports Medicine (ACSM) Guidelines for Exercise and Cancer for improving HRQoL [8]. The exercise prescription will be individualized by the trial AEP based on the participants’ goals, exercise history and preferences, baseline testing results, and health status, including location/extent of bone metastases. All participants will be asked to maintain customary physical activity and dietary patterns over the intervention period (apart from the programmed exercise).

Prior to each supervised session, the trial AEP will measure the participants’ heart rate (HR) and blood pressure to identify potential contraindications to commencing exercise, as outlined by the ACSM [40]. Each session will commence with a 5 min warm-up comprising low-impact aerobic activities, such as stationary cycling, at a progressive very light/light intensity (rating of perceived exertion (RPE) of 9–10 on the 6–20 Borg Scale), and conclude with a 5 min cool-down period of stretching activities.

The 20 min aerobic exercise component will comprise 60–85% peak HR (HR_peak_) (equivalent to RPE 12–15; Polar Electro Oy, Kempele, Finland), interspersed with 2 × 4 min bouts of high intensity (>85% HR_peak_; equivalent to RPE 16–19) exercise. HR_peak_ from the baseline cardiopulmonary exercise test (CPET) will be used to calculate the target intensity if the criteria for achieving maximal oxygen uptake (V̇O_2max_) is met. Where the baseline CPET result does not meet the criteria for a V̇O_2max_ result, i.e., peak oxygen uptake (V̇O_2peak_) or an invalid test, HR_peak_ will be determined using the equation [41]:

HR_peak_ = 208 − (0.7 × age in years)

The resistance exercise and impact loading prescriptions will be guided by the protocols for people with bone metastases used by Hart et al. [42] and Rief et al. [43]. Specifically, the resistance training component of the program will include exercises that target the major trunk, upper, and lower body muscle groups, modified based on the location and extent of bone lesions and bone pain [42]. Intensity and volume of resistance exercise will involve 2–3 sets of 6–10 repetition maximums (RMs) (i.e., the maximal weight that can be lifted 6 to 10 times), with 30–60 s rest between sets. Target intensity will be 60–85% (6–9 on the OMNI-Resistance Exercise Scale of Perceived Exertion (OMNI-RES) [44]. To ensure the progressive nature of the training program, participants will be encouraged to work past the specific RMs prescribed. The resistance will be increased by a 5–10% increment for the next set/training session if the participant is able to perform more repetitions than the RMs specified during a set.

Bone loading activities will progressively increase across the intervention from 40 impacts per session to 100 impacts per session for non-affected skeletal sites [45]. Participants with pelvic, axial skeleton (lumbar), or lower limb bone lesions will be prescribed isometric exercise targeting the spine and pelvis in positions of support, e.g., supine, prone, or seated for unstable lesions. For participants without the aforementioned bone lesions, the intensity of impacts, based on the ground reaction forces produced, will be progressively increased across the intervention, i.e., level 1: isometric exercise targeting spine and/or pelvis; 2: marching; 3: stomping; 4: jumping; 5: drop jump from height (range 10–30 cm) [45]. Progression will account for any pre-existing musculoskeletal injuries and/or pain. Participants will be instructed to land on their heels with approximately 5° knee flexion on impact. One minute rest periods will separate jump sets with a minimum of four sets of 20 impacts per set. Unilateral exercises, i.e., marching, stomping, will have a minimum of 80 impacts per leg (4 × 20 impacts per leg), with a minimum of 80 total impacts for bilateral exercises, i.e., jumping, drop from height.

Exercise sessions will be cancelled/postponed if the participant experiences a fever (>38.0 °C), infection, platelet count <50 × 10^9^/L, hemoglobin <90 g/L, higher grade cardiac arrhythmias, or life-threatening clinical complication. Neutropenia of any grade without fever will not be considered a contraindication to exercise training.

#### Stepped Down Supervision Approach

The modular multi-modal exercise program should be undertaken three times per week. For the first twelve weeks, two exercise sessions per week will be supervised by an AEP and once at home. For home-based sessions, elastic resistance bands will be provided, with advice for the purchase of dumbbells of appropriate weight. High intensity training will be replaced by a moderate intensity walk three times per week if a bike or treadmill is unavailable. Exercise supervision in the following three months will be stepped down to home-based sessions, with an optional weekly group class (2 to 6 participants) supervised by the study AEP. In addition, the AEP will telephone participants every two weeks to check on their adherence, any adverse events, prescribe progressions/regressions as appropriate, and answer any questions. Motivational interviewing will be used if any barriers are expressed. Following the first six months, all participants will be encouraged to maintain three exercise sessions per week for a further six months without group sessions. At the completion of their study involvement, participants will receive education regarding the Australian Government Department of Health Chronic Disease Management Plan, with no direct referrals to exercise physiologists or group classes.

## 3. Outcome Measures

The data and sample collection schedule is depicted in Figure 1, and detailed in Table 1. All testing sessions will be conducted in the research laboratories within the School of Human Movement and Nutrition Sciences at The University of Queensland and will be completed by two researchers, consistently assigned to the same tasks. Prior to each testing session, participants will be asked to: (i) maintain a hydrated state in the 24 h prior to testing; (ii) fast, including abstaining from caffeine and alcohol intake for 8 h prior to testing; and (iii) avoid any vigorous, high or unaccustomed moderate intensity physical activity for the preceding 24 h. Wherever possible, the testing session will be scheduled for the morning hours, with all subsequent testing timepoints scheduled at the same time of day for each participant. Prior to the baseline testing session, participants will be required to record a 3-day diet diary, which they will be required to replicate prior to all subsequent testing sessions. Participant adherence to the testing standardization measures will be evaluated prior to the commencement of each assessment. If testing standardization measures are not met, the researcher will confirm the participant’s understanding of the pre-testing requirements and reschedule the assessment for another day. Inter- and intra-tester reliability will be determined for data-collecting investigators for all outcome measures as appropriate. Assessments will be performed in a consistent order with appropriate rest periods to minimize testing fatigue. Any alterations in medical status or medications, hospital admission, or concomitant study participation will be documented for future reference during analysis of results.

### 3.1. Primary Outcome

#### Cancer-Specific Quality of Life (EORTC QLQ-C30 and QLQ-MY20)

The primary outcome of this study is HRQoL after the 3-month supervised exercise intervention compared with the waitlist control. HRQoL will be measured using the overall HRQoL summary score for the core HRQoL questionnaire of the European Organisation for Research and Treatment of Cancer Quality of Life Questionnaire (EORTC QLQ-C30, version 3.0) [46]. The EORTC QLQ-C30 is a widely used and well-validated cancer-specific instrument [3] with good psychometric properties in solid tumors [47,48]. It has acceptable reliability and validity in MM (Cronbach’s α = 0.70–0.92, except role function = 0.54), with an excellent ability to discriminate between patients in different objective disease states and a high responsiveness to changes in patients’ clinical status over time [3,49]. In a recent Delphi analysis, consensus was reached to collect patient-reported outcomes using the EORTC QLQ-C30 in patients with MM [50]. The minimal important difference (MID), defined as the smallest change in a HRQoL score considered important to patients that would lead the patient or clinician to consider a change in therapy, will be based on those estimated for individual scales of the QLQ-C30 in patients with MM [51].

The EORTC QLQ-C30 summary score encompasses all symptom (e.g., fatigue, pain) and function domains (e.g., emotional and social functioning) assessed by the QLQ-C30. The summary score is a strong prognostic factor for overall survival above and beyond that provided by clinical and sociodemographic variables for patients with MM [7]. The summary score also appears to have more prognostic value than the global QoL, physical functioning, or any other scale within the QLQ-C30 [7].

The European Organisation for Research and Treatment of Cancer Quality of Life Questionnaire Multiple Myeloma module (EORTC QLQ-MY20) is a reliable and valid instrument, recommended as a supplement to the QLQ-C30 instrument in patients with MM (Cronbach’s α = 0.70–0.92) [52]. As with the QLQ-C30, QLQ-MY20 domain scores are averaged and transformed linearly to a score ranging from 0–100. A high score for disease symptoms and side effects of treatment represents a high level of symptomatology or problems, whereas a high score for future perspective and body image represents better outcomes.

### 3.2. Secondary Outcomes

#### 3.2.1. Cardiorespiratory Fitness (V̇O_2peak_ and Oxygen Efficiency Uptake Slope)

A cardiopulmonary exercise test (CPET) will be conducted to determine V̇O_2peak_, which represents a strong predictor of decreased total cancer mortality risk independent of adiposity [53]. This measure has been found to be feasible and safe in a sample of patients with MM greater than six months post autologous stem cell transplantation and in remission [54]. The oxygen uptake efficiency slope (OUES), a submaximal index of cardiorespiratory reserve, will also be determined [55]. OUES has been shown to correlate with V̇O_2peak_ in older patients with colorectal cancer [56], indicating that it as a valid index for exercise tolerance. The exercise intensity (submaximal or maximal) does not affect OUES values [57]; hence, it is used in clinical conditions which prevent patients from being able to perform a maximal CPET. Additional parameters that will be determined from the CPET include test duration as a measure of exercise capacity and V̇O_2_ and power output at ventilatory threshold as measures of submaximal aerobic capacity.

V̇O_2peak_ testing will be conducted under medical supervision, with electrocardiography continually monitored throughout the protocol. All procedures will be carried out in accordance with the exercise testing criteria defined by the ACSM [40]. Prior to commencement, participants will be checked for any contraindications to exercise testing, including blood pressure measurement.

All V̇O_2peak_ testing will be completed using a cycle ergometer (Lode Rehcor, Lode B.V., Groningen, Netherlands). The V̇O_2peak_ test will require participants to progressively cycle to volitional fatigue; oxygen uptake (V̇O_2_) and carbon dioxide production (V̇CO_2_) will be measured continuously during exercise. Using a stationary metabolic cart system (COSMED Quark CPET, Rome, Italy), expired air will be analyzed for fractional expired oxygen (FEO_2_) and fractional expired carbon dioxide (FECO_2_) every 15 s during exercise from a mixing chamber, while minute ventilation will be recorded every 15 s using a turbine ventilometer. Gas analyzers will be calibrated immediately prior to testing and validated after each test using a certified beta gas mixture (BOC, Brisbane, Australia). The ventilometer will be calibrated before each test using a 3 L syringe (Hans Rudolph Inc., Shawnee, PA, USA) in accordance with the manufacturer’s instructions. V̇O_2peak_ will be recorded as the mean of the two highest consecutive 30 s V̇O_2_ readings, with a maximal test determined if the two highest readings demonstrate a plateau. The test will be determined to be invalid if it is terminated prior to reaching a ventilatory threshold. Peak power output will be recorded as the highest 30 s power output completed during the test.

The testing protocol, modified from Wasserman and Whipp [58], will begin with three minutes of rest for respiratory normalization, followed by four minutes of warm-up at no or very low resistance. Thereafter the electronic resistance provided by the cycle ergometer will increase incrementally by 10–15 watts each minute. To ensure the test lasts between 10 and 12 min, the researcher may use their clinical judgement to adjust the wattage for the warm-up and the test increments for participants with very low or very high predicted cardiorespiratory fitness. If adjustments to the warm-up wattage and increments are made, these will be replicated at all subsequent V̇O_2peak_ tests for that participant. Participants will be asked to maintain a cycling cadence between 60 and 70 revolutions per minute throughout the test.

Throughout the V̇O_2peak_ test, HR, blood pressure, and RPE (6–20 Borg scale) will be monitored. Blood pressure will be monitored every two minutes, whilst HR will be continuously monitored throughout the V̇O_2peak_ test using the COSMED automatic blood pressure cuff. Participants’ RPE will be recorded in the final 20 s of each minute. The test will be terminated when participants reach volitional fatigue, when participants choose to terminate exercise, or at the discretion of the researchers, in accordance with the indications for exercise test termination as outlined by the ACSM [40]. HR recovery will be monitored for 5 min immediately following the V̇O_2peak_ test, with participants only discharged from the laboratory once their blood pressure and HR return to within 10–15 mmHg and 15 bpm of pre-exercising levels.

#### 3.2.2. Body Composition, Bone Mineral Density, and Bone Architecture

Dual energy x-ray absorptiometry (DXA, Hologic Horizon A, Waltham, MA, USA) will be used to assess bone mineral density (whole body, lumbar spine, and hip) and body composition (regional and whole body fat and fat-free mass), allowing the assessment of the metabolic status of bone and identification of the risk level for future fractures [59]. Bone architecture will be assessed by peripheral quantitative computed tomography (pQCT; XCT-3000, Stratec Medizintechnik GmbH, Pforzheim, Germany), a non-invasive low radiation method for assessing three-dimensional bone microarchitecture and volumetric bone mineral density in cortical and trabecular compartments of the femur and distal radius [60]. Scans of the skeletally non-dominant leg and forearm will be performed with skeletal dominance determined from functional dominance. Femur and radial length will be measured to the nearest millimeter with an anthropometric ruler. Femur length will be measured as the distance from the apex of the lateral epicondyle to the inguinal crease. Radial length will be measured as the distance from the tip of the radial head to the tip of the radial styloid process. Two image slices of the femur (4% and 33% of femur length proximal to the distal endplate) and two image slices of the radius (4% and 66% of radial length proximal to the distal endplate) will be obtained. The scan parameters will be voxel size, slice thickness, and scan speed of 0.5 mm, 2.3 mm, and 20 mm/s, respectively. A planar scout view over the joint line will be acquired in order to place the anatomic reference line. Analyses will be conducted using host software (Version 6.20, Stratec Medizintechnik GmbH, Pforzheim, Germany).

#### 3.2.3. Anthropometry

Body mass index (BMI) and waist–hip ratio will be measured according to the international standards for anthropometric assessment [61]. Body mass and stature will be measured using an electronic, calibrated scale (A & D Mercury, Pty Ltd., Thebarton, Australia) and standard stadiometer (Seca, Birmingham, UK), respectively. These measures will be used to calculate BMI and to track changes in body mass. Waist circumference will be measured using an anthropometric steel tape measure (Lufkin W606M retractable steel tape; Cooper Tools) at the narrowest point between the lowest costal border and iliac crest, while hip circumference will be measured at the level of greatest posterior gluteal protuberance. These measures will be used to calculate the waist–hip ratio and to track changes in the waist circumference.

#### 3.2.4. Neuromuscular Strength and Balance

##### Grip Strength

Grip strength will be used as an indicator of upper extremity body strength and assessed for the dominant and non-dominant hands using a spring-resistance dynamometer (TTM Advanced Analogue Hand Grip Dynamometer, Tokyo, Japan), as previously described [62]. Grip strength is an independent predictor of mortality in older adults and can potentially identify patients, including those with a high level of function, who are at risk of deteriorating health [63].

##### Leg Neuromuscular Power

The 30 s sit-to-stand (30STS) test is used as a measure of lower limb neuromuscular power, as it involves activation of multiple muscles of the lower limb, most notably the gluteal and knee extensor (quadriceps femoris) muscles [64]. It is also able to assess the fatigue effect caused by the number of sit-to-stand repetitions. In a population of well-functioning 70- to 79-year-olds, those with sit-to-stand times in the high risk group were more likely to experience adverse health-related events, such as persistent (severe) lower extremity limitation, death, and hospitalization (hazard ratio = 1.59, 95% CI: 1.41, 1.78 (*p* < 0.001)) [65]. Participants will be required to stand up and sit down on a standard armless chair as quickly as safely possible for 30 s. Participants will fold their arms across their chest and be instructed to stand up completely and make firm contact when sitting. Timing will begin on the command “go” and cease after 30 s. Only one test trial will be performed to minimize fatigue.

##### Isometric Mid-Thigh Pull

Given the high proportion of bone lesions in people with MM, the isometric mid-thigh pull will be used as a measure of body strength. Strength measured via the isometric mid-thigh pull has been shown to correlate with overall body strength [66] and has been used in cancer populations to determine responses to concurrent resistance training and high intensity interval training [67]. It requires little familiarization and can be implemented across a range of populations, including older adults. All measures will be performed using a custom isometric testing device that includes a step-wise adjustable bar and a portable force plate for measuring ground reaction forces (Kistler, Ostfildern, Germany) [68], with sampling at 1000 Hz from the vertical axis using native software (Kistler Bioware 5.3.2.9, Ostfilferm, Germany). The peak or highest force output will be recorded and used as a measure to indicate maximal lower limb muscle strength.

##### Y-Balance Test

The Y-Balance Test (YBT) is a simple, reliable tool to measure dynamic balance. It has a high level of test–retest reliability and has been shown to have strong relationships with knee flexor and hip abductor strength [69]. The test measures the participant’s strength, stability, and balance on one leg whilst simultaneously reaching as far as possible in three separate directions (anterior, posterolateral, and posteromedial) with the other leg. The YBT composite score is calculated by summing the three reach directions and normalizing the results to the lower limb length [70]. The ability to balance on each leg in a static stance will be assessed prior to completion of the YBT to indicate any safety concerns. A duration of >30 s will be classified as successful test completion.

#### 3.2.5. Pain and Bone Pain

Pain will be assessed via the Brief Pain Inventory (BPI), previously known as the Brief Pain Questionnaire, a self-administered questionnaire that was originally designed to assess cancer pain. Test–retest reliability has been assessed for malignant pain and shows good reliability for pain intensity (Cronbach’s α = 0.8) and pain interference (Cronbach’s α = 0.8) [71]. Internal consistency is high for the severity scale (0.81 < α < 0.89) [72] and interference scale (0.88 < α < 0.95) [73].

Bone pain will be assessed using the Functional Assessment of Cancer Therapy Bone Pain Subscale Questionnaire (FACT-BP), which has been validated for use in cancer populations [74]. The scale has been shown to be a robust and concise tool for assessing cancer-related bone pain, in addition to the impact of that pain upon functioning and quality of life. The internal consistency reliability coefficients for the questionnaire exceed acceptable standards (Cronbach’s α = 0.93–0.96), with evidence of construct validity. Known-group validity was supported by score shifts in the anticipated direction (Cohen’s d effect size = 0.36).

#### 3.2.6. Cancer-Related Fatigue (FACIT-F)

Cancer-related fatigue will be assessed using the Functional Assessment of Chronic Illness Therapy Fatigue Subscale Questionnaire (FACIT-F). The FACIT-F has been previously reported to have high reproducibility and validity (Cronbach’s α = 0.95–0.96), with good divergent validity to discriminate between levels of disease severity and functional status [75] and hemoglobin (used as a measure of fatigue) [76]. The FACIT-F has 43 items in total and includes five subscales: physical, functional, social/family, emotional well-being, and a fatigue-specific domain.

#### 3.2.7. Functional Disability (Oswestry Low Back Pain Disability Questionnaire)

The Oswestry Low Back Pain Disability Questionnaire (OLBPDQ) will be used to measure permanent functional disability. The test is considered the “gold standard” of low back functional outcome tools. It is a unidimensional scale with overall excellent construct validity, good internal consistency (Cronbach’s α = 0.85), and the ability to discriminate the severity of functional disability [77].

#### 3.2.8. Myeloma-Specific Patient Reported Outcomes (MyPOS and FACT-MM)

Myeloma-specific patient reported outcomes will be assessed using the Myeloma Patient Outcome Scale (MyPOS) and the Functional Assessment of Cancer Therapy Multiple Myeloma subscale Questionnaire (FACT-MM). The MyPOS is a 30-item, myeloma-specific version of the Palliative Care Outcome Scale. It was developed through a qualitative investigation of the issues most important to the HRQoL of people with MM and is a valid and reliable tool for use in the routine clinical care of myeloma patients (Cronbach’s α = 0.89) [78]. Factor analysis confirmed three subscales; (i) symptoms and function (14 items covering physical symptoms and functional impairments); (ii) emotional response (8 items describing the emotional impact of the disease); and (iii) healthcare support (5 items on information needs and satisfaction with healthcare). Higher scores represent worse HRQoL.

The FACT-MM [79] has 14 items which are a disease-specific, patient-reported outcomes measure for the assessment of HRQoL among people with MM. Participants will indicate how true the scale items are for them on a scale from 0 (not at all) to 4 (very much).

#### 3.2.9. Falls Self-Efficacy (Falls Efficacy Scale—International (FES-I))

The Falls Efficacy Scale—International assesses the level of concern about falls during 16 activities of daily living, ranging from basic to more demanding activities, including social activities, which may contribute to quality of life. It was originally developed to assess concern about falls in older people [80]. The FES-I has excellent reliability and validity with regard to external physiological and neuropsychological measures [80,81]. The level of concern for each item is scored on a 4-point scale (1 not at all, 2 somewhat, 3 quite a lot, 4 very), with the total score ranging from 16 to 64.

#### 3.2.10. Blood Biomarkers

A qualified and experienced phlebotomist will complete all blood collection procedures. Blood (24 mL) will be sampled from an antecubital vein using a 23-gauge needle and collected into 4 × 6 mL plastic vacutainers (BD), uncoated for serum collection (1 × 6 mL) and coated with anticoagulant agent K_2_EDTA (2 × 6 mL) and lithium heparin (1 × 6 mL) for plasma. Plasma samples will be immediately stored on ice, whilst serum will be allowed to clot at room temperature for 60 min before centrifugation at 3000 rpm for 10 min (approximately 900× *g*) at −4 °C. Plasma and serum samples will be pipetted into 0.3 mL aliquots, frozen at −80 °C, and stored for subsequent analyses.

##### Adipokines

Bone marrow adipose tissue (BMAT) interrelates with bone marrow cells and other immune cells, and several studies have identified BMAT as a key driver in MM progression [82,83,84,85]. BM adipocytes isolated from MM subjects have been shown to increase myeloma growth in vitro and may preserve cells from chemotherapy-induced apoptosis [82]. They may have a role in MM progression, bone homing, chemoresistance, and relapse, due to local endocrine, paracrine, or metabolic factors [83,84,85]. Preclinical results indicate that modifying adipose tissue and BMAT could be a successful MM therapy [86]. Exercise can alter BMAT [87] and can alter the secretion by adipocytes of numerous adipokines [88]. The levels of plasma total and high molecular weight adiponectin will be measured by enzyme-linked immunosorbent assay.

##### Bone Health

Markers of bone resorption (C-terminal (CTX) telopeptide of type 1 collagen) and formation (alkaline phosphatase (total and bone specific), procollagen type 1 N-terminal propeptide (P1NP)) will be measured by enzyme-linked immunosorbent assay.

##### Immune Function

Specific markers of immune function will be assessed using flow cytometry. As a general marker of inflammation, high-sensitivity C-reactive protein will be measured by immunoturbidimetric assay. Using multiplexed bead-based immunoassay, an inflammatory cytokine panel of 16 markers (IL-1beta, IL-2, IL-4, IL-5, IL-6, IL-8, IL-10, IL-13, TNF-alpha, G-CSF, MCP-1, GM-CSF, IFN-alpha, IFN-gamma, IL-17A, IL-12p40) will be analyzed.

##### Metabolomic and Lipidomic Analyses

Untargeted metabolomic and targeted plasma lipidomic profiles will be determined using liquid chromatography-coupled tandem mass spectroscopy analysis [89,90]. In addition, extracellular vesicles will be isolated from plasma for untargeted proteome and targeted lipidome analyzed by liquid chromatography-coupled tandem mass spectroscopy [91]. Since changes in the serum metabolomic profile and extracellular vesicles have been observed in MM patients after remission [92] and associated with progression [93], the disease and treatment status of patients will be noted before interpretation.

#### 3.2.11. Self-Reported and Objective Physical Activity

The Godin Leisure Time Physical Activity Questionnaire (LTPAQ) will be used to track the self-reported physical activity behaviors [94]. The Godin LTPAQ requires participants to recall during a typical seven day week the frequency and duration of leisure-time physical activity completed at three separate intensities: mild, moderate, and vigorous intensity. The Godin LTPAQ has been shown to have a modest correlation compared with accelerometry-derived measures of physical activity of at least moderate intensity in cancer survivors (r = 0.46) [95] and demonstrates high test–retest reliability (r = 0.75) [96].

Objective physical activity will be measured using the Actigraph wGT3X-BT, a valid, inclinometer/accelerometer that measures time spent sitting, standing, and stepping (overall and at a light or moderate-to-vigorous physical activity intensity) [97,98,99]. Participants will be asked to wear the device on an elastic waist belt aligned with the right anterior axillary line during waking hours for seven days after each testing session. Participants will be asked to complete a wear-time log and activity diary during the monitoring period, recording wake and sleep times, and, if the monitor was removed, the duration and reason (showering, bathing, swimming, sleeping, or engaging in contact sport). Accelerometers will be initialized with 30 s epochs and a 30 Hz sampling frequency. A valid day will be defined as a minimum wear-time of 10 waking hours, with non-wear-time defined as 60 min or more of consecutive activity counts of zero. For activity data to be included, participants must satisfy a minimum wear-time criterion of at least four of the seven days, with at least one of the days being a weekend day [100]. ActiGraph wGT3X-BT data will be processed using R-Package GGIR [101] to calculate time in sedentary (<30 mg), light (30–99 mg), moderate (100–399 mg), and vigorous (400+ mg) PA using the widely recognized thresholds proposed by Hildebrand et al. [102]. Additionally, sedentary behavior and moderate-to-vigorous PA will be estimated in bouts of 30 min and 1–5, 5–10, and >10 min, respectively.

#### 3.2.12. Enjoyment (Physical Activity Enjoyment Scale-8 (PACES-8)

A shortened version of the physical activity enjoyment scale (PACES-8) will be administered by the trial AEP at the conclusion of the supervised training sessions at 4-weeks, 8-weeks, and 12-weeks to assess participant enjoyment. This is a simplified version of the physical activity enjoyment scale, as originally described by Kendzierski and DeCarlo [103], with strong psychometric integrity, and it has been validated in older populations [104]. The PACES-8 consists of 8 subscales that each relate to an aspect of enjoyment. Along a 7-point continuum for each subscale, participants are asked to provide a rating to reflect their agreement with one of two bi-polar statements at each end of the continuum (e.g., It’s no fun at all, It’s a lot of fun). The PACES-8 has been shown to have good internal reliability coefficients at two timepoints six months apart (McDonald’s ω = 0.93, 0.93) [104] and correlated positively with experienced physical change (r = 0.42, 0.47), psychological/emotional change (0.41, 0.42), and functional change (0.39, 0.29) at timepoints 1 and 2, respectively.

### 3.3. Tertiary Outcomes

#### 3.3.1. Qualitative Analysis

To gain an in-depth understanding of the experiences of participants and the perceived benefits and barriers of the intervention, semi-structured interviews will be conducted. An interviewer will conduct 45 min face-to-face interviews with participants after the completion of the 3-month unsupervised exercise intervention. A standard script will be used. Interviews will be transcribed and analyzed in NVivo 12 Plus (QSR International, Chadstone, Australia) using the constant comparison coding method to reduce the data to themes using a qualitative descriptive approach. Codes will be assigned to salient text segments across the entire data set, then combined to define overarching themes. Two researchers will develop a descriptive coding frame based on *a priori* concepts and will work collaboratively to refine the coding frame and develop concordance about the scope and content of each code. This coding frame will provide a “start list” to which inductive codes will be added to capture emergent concepts during this and the subsequent coding process [105].

Initial themes will be developed by reading within and across the codes to look for conceptual patterns and by synthesizing the points that are most relevant to the research questions [105]. Themes will be further refined through this reflective process, with review of the transcripts and audio recordings to check the validity of assertions. Throughout this process, analysis will involve reviewing data against each other to identify conceptual differences/similarities and degrees of prevalence and strength.

#### 3.3.2. Feasibility and Adherence

The feasibility of the program will be measured by the rates of eligibility, recruitment, attrition, attendance (defined as: n _sessions attended_ × n _sessions prescribed_^−1^ × 100), and adherence to the program intensity and duration for each component of the intervention, i.e., aerobic, resistance and bone loading.

Attendance at the supervised sessions will be recorded by the supervising AEP, in addition to the self-reported completion of the weekly home-based session. Adherence records will be completed by the supervising AEP at each supervised session with detailed reasons for non-adherence recorded. Adherence to the aerobic component of the protocol will be defined as: minutes at the workload prescribed by the AEP, i.e., 12 min at moderate-to-vigorous intensity (60–85% HR_peak_) and 8 min at high intensity (>85% HR_peak_). Adherence to the resistance training component of the protocol will be defined as: minimum of 80 repetitions (5 exercises, 2 sets, 8 repetitions = 5 × 2 × 8) at a minimum intensity of 5/10 on the OMNI-RES scale [44]. Adherence to the bone loading component of the protocol will be defined as: minimum of 80 repetitions. The minimum intensity for adherence will be 2, where 1: isometric exercise targeting spine and/pelvis; 2: marching; 3: stomping; 4: jumping; 5: drop jump from height (range 10–30 cm). Unilateral exercises, i.e., marching, stomping, will have a minimum of 80 impacts per leg (4 × 20 impacts per leg), with a minimum of 80 total impacts for bilateral exercises, i.e., jumping, drop jumping from height.

*Apriori* cut points were defined to determine successful adherence to each component of the exercise program. Cut-off values for the feasibility criterion (a recruitment rate of ≥25% [106]; an attrition rate of <25% [107]; and attendance and adherence of ≥75% [108]) were established *a priori* as clinically relevant based on previous studies in other cancers.

#### 3.3.3. Adverse and Serious Adverse Events

Safety of the intervention will be measured through reporting adverse and serious adverse events. The trial AEP will record at each testing and supervised training session whether an adverse event has occurred and, if so, the AEP will provide details to the governing ethics committee. Events at unsupervised sessions will require self-report by the participant to the trial AEP. Adverse events will be reported to the study clinicians and the participant’s treating doctor for further decisions on safety and any required investigations. Trial insurance provided through the sponsor, The University of Queensland, includes provision for post-trial care if required.

## 4. Data Collection, Management and Monitoring

All data from the study participants will be collected using a case report form designed for the standardization of data collection (available on request). Electronic copies of participant data, audio recordings, and transcripts of interviews will be stored in various modes (computer hard drive; external hard drive; and cloud-based systems), all of which will be de-identified and password protected, maintaining confidentiality at all times. Electronic data repositories will only be accessible by the study investigators and associated data analysts. All hard-copy files will be stored in a securely locked filing cabinet at the School of Human Movement and Nutrition Sciences, The University of Queensland. Disposal of hard-copy files will be performed in accordance with general procedures, the regulations of the administering ethical board and The University of Queensland.

Results will be made available to participants (including any publications that result from the project) upon individual request following the completion of the study. Individual participants will not be identified in any resultant manuscripts or reports of this trial.

The study investigators will permit study-related monitoring, audits, and inspections of all study related documents and facilities by the governing ethics committees. An independent data monitoring committee is not needed, given the role of the governing ethics committees.

## 5. Statistical Considerations and Data Analyses

A priori power calculations determined that a sample of 44 participants (*n* = 22 per group) will be needed to detect a mean difference of 6.7 points [51] between groups on the EORTC QLQ-C30 MY20 after the 3-month supervised exercise phase, with 80% power and 5% alpha. To account for an anticipated dropout rate of 25%, 60 participants will be recruited to the study. A difference of 6.7 on the physical function subscale of the EORTC QLQ-C30 MY20 questionnaire, determined by receiver operating characteristic curve analysis, corresponds to the minimum clinically meaningful change considered important by people with MM [51].

Data will be analyzed using the Stata statistical software package (version 15.0, StataCorp, College Station, TX, USA). Data entry will be performed in duplicate, and accuracy will be assessed with automatic cross-checking procedures. Range checks for data values will be performed to promote data quality. Data will be assessed for normality using the Shapiro–Wilk test. Subsequent analyses will include standard descriptive statistics, *t* tests, correlation, regression, and one-way repeated measures ANOVA or the comparable non-parametric test as necessary to examine differences among timepoints. Each model will include covariates, as appropriate, and will be adjusted using the baseline value as a fixed continuous covariate. For accelerometer data, means and 95% confidence intervals will be calculated and repeated measures accounted for across multiple days within participants. Quality of life questionnaire items will be analyzed using random effects mixed modelling with Fisher’s least significant difference tests for post hoc analyses to compare mean changes in outcomes between groups at each assessment period, with time and group as the fixed factors. A time × group interaction term will be used to formally test for differences between groups (alpha = 0.05).

The number of participants who provide data at each timepoint will be reported, with reasons as to why participants did not attend. Incomplete data and missing values will primarily be managed using intention-to-treat analyses, using a 70% cut-point for adherence. To ensure the robustness of the findings, a secondary sensitivity analysis will be performed [109]. Non-continuous data will be analyzed using multinomial logistic regression. Metabolomics and lipidomics data will be evaluated using multivariate statistics, including principal component analysis and sparse partial least squares discriminant analysis. The blood molecular time-series measures will be analyzed to determine the metabolic and immune pathways altered by exercise and to determine biomarkers of responders for physical fitness, fatigue, and pain, if any. Exercise and control groups will be further stratified by therapy and remission duration. All tests will be two-tailed, and an alpha level of 0.05 will be applied as the criterion for statistical significance. There are no interim analyses planned or early trial termination guidelines.

### Cost-Effectiveness Analysis

Cost-utility analysis (CUA) will be used to explore whether the intervention provides health benefits at the individual level. CUA compares the incremental costs and benefits, expressed in quality-adjusted life years (QALYs) gained, of alternative programs. Health utilities will be derived from the EORTC QLU-C10D, a subset of 10 questions from the EORTC QLQ-C30 [110]. Costs will be obtained from a questionnaire that will be completed by the waitlist control and exercise groups prior to commencement of the intervention and during the 3-month supervised and 6-month unsupervised phases to determine individual costs associated with the intervention and other health resource utilization. Costs will be measured from a societal perspective and will include intervention costs, healthcare costs, costs of informal care, sports costs, unpaid productivity costs, and absenteeism costs. Healthcare costs include the costs of primary healthcare (i.e., general practice, physical therapist), secondary healthcare (i.e., outpatient care, hospitalization), and prescribed, as well as over-the-counter medication. The value of the resources consumed will be determined using unit prices from standard costing resources (e.g., Medicare Benefits Schedule, award wage schedules).

Resource utilization during the 3-month supervised and 6-month unsupervised phases will be recorded. This includes labor (e.g., to deliver the intervention and group classes) and non-labor (e.g., gymnasium use), as well as the cost of usual care provided by the hospital. Resource use will be costed at market rates (e.g., industrial award rates for labor costs) for use in cost-effectiveness analyses. The resource use associated with the exercise intervention delivery will include labor time, i.e., AEP hours planning the exercise sessions (e.g., phoning/meeting people to book their session, pre-exercise safety screening), delivering the exercise sessions, and documentation time for clinical notetaking. Time spent for research assessments will not be included. Resource use will also include non-labor costs associated with delivering the intervention from the perspective of the health service, e.g., cost of hiring gym space.

## 6. Ethics and Dissemination

This study has institutional ethical approval through the Human Research Ethics Committees of Greenslopes Private Hospital, Metro South Hospital and Health Service, The University of Queensland, and QIMR Berghofer Medical Research Institute (#18/58 GREC; #HREC/2019/QMS/47400; #2018002644/18/58 UQ HREC; P#2352). Any future amendments to the protocol and/or associated documents will be submitted for approval to the aforementioned Human Research Ethics Committees. Consent for use of stored blood samples and research data for future research studies will also be collected, though any future research will obtain prior approval by a Human Research and Ethics Committee before commencement. The study will be conducted in accordance with the principles of the Declaration of Helsinki of 1975 (revised in 2013), according to international standards of Good Clinical Practice guidelines, applicable Australian government regulations, and Institutional research policies and procedures.

Outcomes of this RCT will be reported in international, high-quality, peer-reviewed journals, and the findings will be presented at national and international scientific conferences and meetings and through State and National representative bodies of exercise oncology and clinical hematology. Findings will also be communicated at community and consumer-led forums and will be presented at local hospital departments and university seminars.

## 7. Discussion

The aim of this study is to determine the impact of an individualized exercise program on patient-reported HRQoL, which has been shown to correlate with survival in people with MM at all disease stages [3,4,5,6,7]. Together with the effects on pain and fatigue, this will provide additional evidence of whether long-term improvements in HRQoL can be maintained whilst access to supervision is decreased. In addition, its effects on cardiorespiratory fitness, bone health, muscle strength, body composition, disease response, and select blood biomarkers will be analyzed. Assessment of changes in cardiorespiratory fitness, neuromuscular strength and power, and body composition, particularly bone and lean mass, will provide empirical evidence for the benefits of the program on the cardiovascular and musculoskeletal health of participants. Measures of bone turnover, adipokines, and immune functioning, as well as metabolomics, will enable investigation of changes in blood parameters that may affect disease survival. Semi-structured qualitative interviews and cost-effectiveness analyses will inform the potential for the program to be implemented as part of standard care for MM.

With the development of complex multi-novel-agent-based regimens for MM, a recent review highlighted the factors of importance to patients with MM in the real-world setting. These included symptom burden, treatment side effects and toxicities, and the ability to continue activities of daily living, including work productivity [111]. It suggested the need for collecting patient-reported outcomes to provide a more holistic definition of the effectiveness of treatment regimens. A strength of our proposed study is that it aligns with these objectives and, with the inclusion of patients at all disease stages, particularly during relapsed disease when symptoms and side effects of therapy may be most pronounced, provides a more comprehensive representation of MM in the clinical setting.

There are several limitations of the proposed study that warrant discussion. Our target population will be limited to those who are able to attend frequent testing and training visits and who are able to obtain an approval to participate from their treating physician. Arguably, those who are frailer, with limited mobility, and who may elicit a greater response from the intervention, will not be included in the study. Whilst the heterogeneous group of people with MM will demonstrate the ability of exercise to elicit improvements across the MM continuum, the study is not powered to conclude its effects within specific disease stages. Finally, the waitlist control group may increase their physical activity, despite being asked to maintain their activity levels for the first 12 weeks, reducing our ability to observe effects of the intervention.

## 8. Conclusions

The proposed study will provide novel evidence for the efficacy of exercise in improving quality of life, as well as providing a comprehensive battery of biophysical and psychosocial measures of health in people with MM. The safety, feasibility, acceptability, efficacy, and cost-effectiveness, as well as maintenance of effects of exercise, will be explored across a 12-month period. The potential findings of this proposed research will ultimately influence the inclusion of exercise as part of standard care to improve the health and longevity of people with MM.

## Figures and Tables

**Table 1:** Schedule of data collection.

	Timepoint			
Assessment or Outcome	Baseline ^a^	3-Months	6-Months	12-Months
*Screening*				
Participant eligibility	✓			
Informed consent	✓			
Doctor’s consent	✓			
Health history and disease status	✓	✓	✓	✓
Demographics	✓			
Concomitant research study participation	✓	✓	✓	✓
*Primary Outcomes*				
Quality of life (EORTC QLQ-C30 and QLQ-MY20)	✓	✓	✓	✓
*Secondary Outcomes*				
Cardiorespiratory fitness (V̇O2peak)	✓	✓	✓	✓
Body composition and bone mineral density (DXA)	✓	✓	✓	✓
Bone architecture (pQCT)	✓	✓	✓	✓
Anthropometry (BMI, waist, and hip circumferences)	✓	✓	✓	✓
Strength (30STS, grip strength, mid-thigh pull)	✓	✓	✓	✓
Balance (Single leg stance, YBT)	✓	✓	✓	✓
Pain and bone pain (BPI, FACT-BP)	✓	✓	✓	✓
Fatigue (FACIT-F)	✓	✓	✓	✓
Functional disability (OLBPDQ)	✓	✓	✓	✓
Myeloma-specific PROs (FACT-MM, MyPOS)	✓	✓	✓	✓
Falls self-efficacy (FES-I)	✓	✓	✓	✓
Blood collection for blood biomarkers, metabolomics, and lipidomics	✓	✓	✓	✓
Self-reported physical activity (Godin)	✓	✓	✓	✓
Sedentary behavior and physical activity (ActiGraph™ accelerometry)	✓	✓	✓	✓
Exercise enjoyment (PACES-8) ^b^		✓		
*Tertiary Outcomes*				
Qualitative analysis (Semi-structured interviews)			✓	
Adherence ^c^		✓	✓	
Safety (Adverse and serious adverse events) ^c^		✓	✓	

^a^ Baseline testing battery is repeated for waitlist control group after three months of usual care. ^b^ PACES-8 survey will be administered at 4-, 8-, and 12-weeks during the exercise intervention. ^c^ Adherence and safety will be monitored continuously throughout the supervised and unsupervised intervention. EORTC QLQ-C30: European Organisation for Research and Treatment of Cancer Quality of Life Questionnaire Core 30; EORTC QLQ-MY20: European Organisation for Research and Treatment of Cancer Quality of Life Questionnaire—Multiple Myeloma module; DXA: Dual energy X-ray absorptiometry; pQCT: Peripheral quantitative computed tomography; BMI: Body mass index; 30 STS: 30-s sit-to-stand test; YBT: Y-Balance Test; BPI: Brief Pain Inventory; FACT-BP: Functional Assessment of Cancer Therapy—Bone Pain; FACIT-F: Functional Assessment of Chronic Illness Therapy—Fatigue; OLBPDQ: Oswestry Low Back Pain Disability Questionnaire; PRO: Patient-reported outcomes; FACT-MM: Functional Assessment of Cancer Therapy—Multiple Myeloma; MyPOS: Myeloma Patient Outcome Scale; FES-I: Falls Efficacy Scale—International; PACES-8: Physical Activity Enjoyment Scale 8.

## Data Availability

This protocol paper does not describe any data. However, the datasets during and/or analyzed during the described study will be available from the corresponding author on reasonable request.

## Supplementary Materials

The following are available online at https://www.mdpi.com/article/10.3390/curroncol29020077/s1, File S1. SPIRIT Checklist.
